# Clinical Utility of Cartridge-Based Nucleic Acid Amplification Test for Tuberculosis and Rifampicin Resistance Detection in the Vindhya Region of India: A Retrospective Study

**DOI:** 10.7759/cureus.114465

**Published:** 2026-08-13

**Authors:** Ramesh P Agrawal, Astha Sinha, Priyanka Chaubey, Pratik N Akhani

**Affiliations:** 1 Microbiology, Government Medical College and Sardar Vallabhbhai Patel District Hospital, Satna, IND; 2 Microbiology, Government Medical College, Seoni, IND; 3 Physiology, GMERS Medical College and Hospital, Dharpur, IND

**Keywords:** drug resistance, india, nucleic acid amplification techniques, rifampin, tuberculosis

## Abstract

Background

Early diagnosis of tuberculosis (TB) and prompt detection of rifampicin resistance are essential to reduce transmission of TB and improve its treatment outcomes. The cartridge-based nucleic acid amplification test (CBNAAT) simultaneously detects the *Mycobacterium tuberculosis* complex and rifampicin resistance within a short turnaround time. This study evaluated the clinical utility of CBNAAT for rifampicin resistance identification and quick microbiological confirmation of extrapulmonary and pulmonary TB in standard clinical practice at a tertiary care center in Central India.

Methods

In this retrospective observational study, records of 2,640 patients with clinically suspected TB who underwent CBNAAT and subsequent line probe assay (LPA) analysis of rifampicin-resistant specimens between June 2024 and May 2025 were analyzed. Demographic characteristics, clinical variables, specimen type, and drug resistance profile were retrieved from laboratory records. Categorical associations were assessed by the chi-square or Fisher’s exact test, and independent associations with CBNAAT positivity were identified by multivariable logistic regression. p < 0.05 was considered significant.

Results

CBNAAT detected *Mycobacterium tuberculosis* in 590 of 2,640 patients (22.35%). Positivity was significantly higher among patients aged 41-60 years, males, rural residents, individuals with diabetes or human immunodeficiency virus (HIV) infection, and those with a history of TB contact or of previous antituberculosis treatment (ATT). Pulmonary specimens showed a substantially higher detection rate (524/1900; 27.58%) than extrapulmonary specimens (66/740; 8.92%). Among confirmed cases, 556 (94.24%) were rifampicin-sensitive, 34 (5.76%) were rifampicin-resistant, and among these rifampicin-resistant cases, 12 (2.03%) were multidrug-resistant (MDR) on LPA. Independent associates of positivity were male sex (adjusted odds ratio (AOR) 1.58, 95% confidence interval (CI) 1.18-2.11), rural residence (AOR 1.42, 95% CI 1.06-1.91), diabetes mellitus (AOR 4.25, 95% CI 3.38-5.34), HIV infection (AOR 5.14, 95% CI 3.57-7.40), pulmonary specimen type (AOR 3.52, 95% CI 2.35-5.27), and previous TB treatment (AOR 2.18, 95% CI 1.61-2.95).

Conclusions

CBNAAT offers rapid microbiological confirmation and thus allows early start of treatment, which is critical in decreasing community transmission of *Mycobacterium tuberculosis*. The significant finding of rifampicin resistance further highlights the utility of this molecular tool to address the challenge of drug-resistant TB (DR-TB) in a tertiary care setting. These findings confirm that the test remains an important part of diagnostic protocols, mainly for pulmonary specimens where the detection rate was substantially higher.

Expansion of molecular diagnostic services and their prioritization as the primary screening tool at all levels of clinical care is recommended to achieve the goals of the National Tuberculosis Elimination Program (NTEP) of India. Higher positivity rates in rural populations indicate the need for policy efforts to improve access to diagnostic facilities in rural areas. Longitudinal studies should be performed in the future to understand the effect of rapid diagnosis by CBNAAT on the long-term outcomes of therapy and mortality rates. Continuous monitoring of drug resistance patterns and integration of molecular testing in routine clinical pathways is important to guide regional TB control strategies.

## Introduction

Despite the availability of effective methods for diagnosing tuberculosis (TB) and a cure, it is still a significant infectious disease in the world today, especially in low- and middle-income countries. According to the World Health Organization (WHO), the total number of TB cases in 2023 was more than 10 million, of which India had the highest burden. Case detection is critical to the success of the End TB Strategy, as timely case detection, early treatment initiation, and interruption of transmission rely heavily on effective case detection [1, 2].

With the increasing occurrence of drug-resistant TB (DR-TB) and multidrug-resistant TB (MDR-TB), the need for fast molecular diagnosis has gained greater relevance. The timely detection of rifampicin resistance has now emerged as a key issue in managing the patient and national control programs, as it is closely linked to multidrug resistance. WHO guidelines currently suggest that rapid molecular assays should be used as the first test for individuals with presumptive TB, as they are able to give rapid bacteriological confirmation as well as detect clinically relevant drug resistance [3].

The cartridge-based nucleic acid amplification test (CBNAAT), based on the GeneXpert Mycobacterium tuberculosis/rifampicin platform, is an automated, single-tube nucleic acid amplification platform that detects the DNA of the *Mycobacterium tuberculosis* complex along with mutations at the rifampicin resistance gene (rpoB) and returns results in about two hours. The simplicity, accuracy, and rapid turnaround results of the CBNAAT make it the preferred first-line molecular investigation for presumptive TB in India's National Tuberculosis Elimination Program (NTEP) [4, 5].

Although CBNAAT is now widely available across India, variation in patient demographics, specimen distribution, and local epidemiology may influence its performance. Published evidence from the Vindhya region of Madhya Pradesh, India, remains limited, particularly for the simultaneous evaluation of pulmonary and extrapulmonary disease alongside rifampicin resistance patterns. Region-specific data are valuable for assessing program effectiveness and identifying factors associated with microbiologically confirmed disease. The present study was therefore undertaken to evaluate the clinical utility of CBNAAT for rifampicin resistance identification and quick microbiological confirmation of TB in standard clinical practice at a tertiary care center in the Vindhya region of India and to examine the demographic and clinical factors associated with CBNAAT positivity.

## Materials and methods

Study design and setting

This retrospective observational study was conducted in the Department of Microbiology, Government Medical College and Sardar Vallabhbhai Patel District Hospital, Satna, Madhya Pradesh, India, and included patients evaluated for suspected TB over a one-year period from June 2024 to May 2025.

Participants

**Figure 1:**
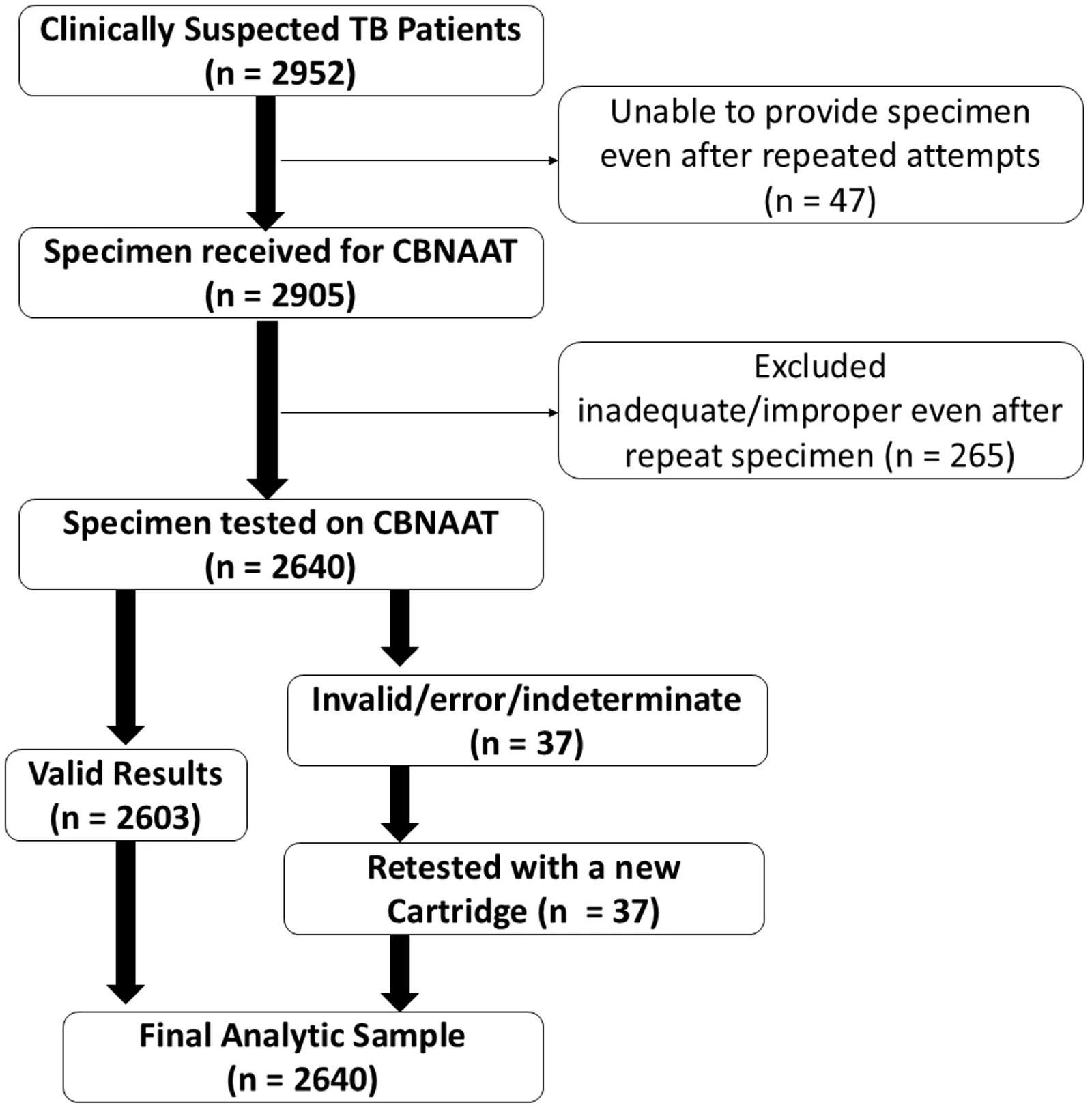
Flow diagram of patient recruitment, specimen processing, and CBNAAT testing TB: tuberculosis; CBNAAT: cartridge-based nucleic acid amplification test

Consecutive patients of all ages and both sexes with clinically suspected pulmonary or extrapulmonary TB who underwent CBNAAT testing under the NTEP during the study period were included. Specimens that were inadequate, improperly collected, or otherwise unsuitable for CBNAAT analysis were excluded. As per NTEP protocol, patients who were unable to produce an adequate sample were asked to submit a repeat specimen. Figure 1 shows the flow of patients through the entire process.

Data collection

Demographic and clinical information was retrieved from laboratory records and hospital case files, including age, sex, place of residence (rural or urban), history of contact with a known TB patient, diabetes mellitus status, human immunodeficiency virus (HIV) status, if any previous treatment was taken for TB, and clinical presentation. A documented close contact with a patient who had been clinically or microbiologically confirmed to have tuberculosis, as documented in the patient's medical records, was considered a history of TB contact. A fasting plasma glucose level of ≥126 mg/dL measured during clinical evaluation or a diagnosis established in the medical records was used to define diabetes mellitus. The findings of NTEP-approved rapid diagnostic tests that were recorded in the hospital records were used to determine the patient's HIV status.

Specimen processing and CBNAAT testing

Pulmonary and extrapulmonary specimens received in the TB laboratory were processed according to the manufacturer’s instructions. CBNAAT was performed on the GeneXpert platform (*Mycobacterium tuberculosis*/rifampicin assay, Cepheid, Sunnyvale, CA, USA), an automated real-time polymerase chain reaction (PCR) assay that detects the *Mycobacterium tuberculosis* complex and rpoB mutations associated with rifampicin resistance, integrating specimen processing, amplification, and detection to provide results within approximately two hours. Specimens positive for *Mycobacterium tuberculosis* were classified as rifampicin-sensitive or rifampicin-resistant; then the rifampicin-resistant specimens were referred for line probe assay (LPA) for confirmation and further drug resistance characterization. In compliance with the manufacturer's instructions and NTEP recommendations, specimens that produced invalid, incorrect, or indeterminate CBNAAT findings were retested using a new cartridge. The final analysis included only the valid test results from subsequent testing.

Outcome measures

The primary outcome was the CBNAAT detection rate of *Mycobacterium tuberculosis* among clinically suspected cases. Secondary outcomes were the distribution of pulmonary and extrapulmonary disease, the proportion of rifampicin-resistant cases and MDR-TB, and the association between CBNAAT positivity and demographic or clinical variables. Instead of formally evaluating diagnostic accuracy against a microbiological reference standard, clinical utility in this study was defined as the capacity of CBNAAT to provide quick microbiological confirmation of *Mycobacterium tuberculosis* and concurrent detection of rifampicin resistance under routine programmatic conditions.

Statistical analysis

Data were analyzed in IBM SPSS Statistics software, version 25.0 (IBM Corp., Armonk, NY, USA). Categorical variables were summarized using frequencies and percentages, and associations were evaluated with the chi-square or Fisher's exact test. Variables with a univariate p-value < 0.20 and clinically relevant covariates, i.e., age, sex, place of residence, history of TB contact, diabetes mellitus, HIV infection, specimen type, and history of previous antituberculosis treatment (ATT), were included in a multivariable binary logistic regression model, and the adjusted odds ratios (AORs) and 95% confidence intervals (CIs) were calculated. Multicollinearity among covariates was assessed using variance inflation factors (VIF), with VIF < 5 taken to be acceptable. A two-sided p-value of < 0.05 was defined as significant.

Ethical approval

The study was approved by the Institutional Ethics Committee of Government Medical College, Satna, Madhya Pradesh, India (Approval No. 136/IEC/GMC/2024) on December 23, 2024. Informed consent was waived because data were anonymized and were extracted via review of routine records only, with no primary data collected from patients.

## Results

A total of 2,640 patients with clinically suspected TB were evaluated by CBNAAT during the study period. The overall detection rate of *Mycobacterium tuberculosis* was 22.35% (590/2,640; 95% CI 20.80%-23.98%), with significantly higher positivity among males, rural residents, patients with diabetes, HIV-positive individuals, and those who had previously received ATT, together with a strong association with a documented history of TB contact. Positivity peaked in the 41-60-year age group (n = 684, 28.65%) (Table 1).

**Table 1:** Demographic and clinical characteristics of the study population with CBNAAT positivity rates TB: tuberculosis; CBNAAT: cartridge-based nucleic acid amplification test; ATT: antituberculosis treatment; HIV: human immunodeficiency virus; χ²: chi-square

Variable	Suspected (n = 2640)	CBNAAT-positive (n = 590)	Positivity (%)	Row % (out of 590 total positive)	χ² statistic	p-value
Age: 1–17 years	502	60	11.95	10.17	32.23	< 0.001
Age: 18–40 years	1142	270	23.64	45.76		
Age: 41–60 years	684	196	28.65	33.22		
Age: >60 years	312	64	20.51	10.85		
Male	1550	398	25.68	67.46	15.41	< 0.001
Female	1090	192	17.61	32.54		
Rural	1532	386	25.2	65.42	10.93	< 0.001
Urban	1108	204	18.41	34.58		
TB contact: present	364	128	35.16	21.69	23.35	< 0.001
TB contact: absent	2276	462	20.29	78.31		
Diabetes: present	372	180	48.38	30.51	91.73	< 0.001
Diabetes: absent	2268	410	18.07	69.49		
HIV: positive	126	72	57.14	12.20	46.27	< 0.001
HIV: negative	2514	518	20.61	87.80		
Prior ATT: received	286	104	36.36	17.63	20.97	< 0.001
Prior ATT: not received	2354	486	20.64	82.37		

Of the 2,640 specimens, 1,900 (71.97%) were pulmonary and 740 (28.03%) were extrapulmonary. CBNAAT detected TB in 524 pulmonary specimens (27.58%; 95% CI 25.62%-29.63%) but in only 66 extrapulmonary specimens (8.92%; 95% CI 7.07%-11.19%), indicating a substantially higher diagnostic yield for pulmonary specimens (Table 2).

**Table 2:** Distribution of pulmonary and extrapulmonary specimens and CBNAAT positivity rates CBNAAT: cartridge-based nucleic acid amplification test; χ²: chi-square

Specimen	Total samples	CBNAAT positive	Positivity (%)	χ² statistic	p-value
Pulmonary	1900	524	27.58	73.06	< 0.001
Extrapulmonary	740	66	8.92		
Total	2640	590	22.35		

Among the 590 confirmed cases, most were rifampicin-sensitive. Rifampicin resistance was detected in 34 cases (5.76%; 95% CI 4.15%-7.94%); of these, 12 patients (2.03%) met the criteria for MDR-TB on LPA; no case of extensive DR-TB was identified (Table 3).

**Table 3:** Distribution of drug resistance among CBNAAT-positive cases TB: tuberculosis; CBNAAT: cartridge-based nucleic acid amplification test; LPA: line probe assay

Drug resistance pattern	Number	Percentage (%)
Drug-sensitive TB	556	94.24
Rifampicin-resistant TB	34	5.76
Multidrug-resistant TB (LPA)	12	2.03

On multivariable logistic regression, male sex (AOR 1.58, p = 0.002), rural residence (AOR 1.42, p = 0.019), diabetes mellitus (AOR 4.25, p < 0.001), HIV infection (AOR 5.14, p < 0.001), pulmonary specimen type (AOR 3.52, p < 0.001) and previous TB treatment (AOR 2.18, p < 0.001) were independently associated with CBNAAT positivity (Table 4). VIF values ranged from 1.01 to 7.35 across all covariates. Except for age (VIF 7.35) and history of TB contact (VIF 5.89), all other covariates showed VIF < 2.1 (sex 1.56, residence 2.01, diabetes 1.25, HIV 1.37, specimen type 1.01, previous ATT 1.46), suggesting no meaningful collinearity among these predictors.

**Table 4:** Multivariable logistic regression of factors independently associated with CBNAAT positivity TB: tuberculosis; CBNAAT: cartridge-based nucleic acid amplification test; HIV: human immunodeficiency virus; AOR: adjusted odds ratio

Variable	AOR	95% CI	p-value
Male sex	1.58	1.18–2.11	0.002
Rural residence	1.42	1.06–1.91	0.019
Diabetes mellitus	4.25	3.38–5.34	<0.001
HIV infection	5.14	3.57–7.40	<0.001
Pulmonary specimen	3.52	2.35–5.27	<0.001
Previous TB Treatment	2.18	1.61–2.95	<0.001

Among the 740 extrapulmonary specimens, lymph node aspirate showed the highest positivity rate (24/120, 20%), followed by pus/abscess aspirate (10/70, 14.29%) and bone/joint aspirate (2/25, 8%). Pleural fluid was the most commonly submitted specimen type among all (260/740, 35.14%), but it showed a lower positivity rate (20/260, 7.69%). CBNAAT positivity rates varied significantly across extrapulmonary specimen types (Fisher's exact test, p < 0.001) (Table 5).

**Table 5:** Distribution of extrapulmonary specimens and CBNAAT positivity rates CBNAAT: cartridge-based nucleic acid amplification test; FNAC: fine needle aspiration cytology; CSF: cerebrospinal fluid

Extrapulmonary specimen type	Total samples	CBNAAT positive	Positivity (%)	p-value
Lymph node aspirate/FNAC	120	24	20	< 0.001
Pleural fluid	260	20	7.69	
CSF	80	4	5	
Ascitic fluid	60	3	5	
Pus/abscess aspirate	70	10	14.29	
Bone/joint aspirate	25	2	8	
Tissue biopsy	60	2	3.33	
Gastric aspirate	40	1	2.5	
Other	25	0	0	
Total	740	66	8.92	

## Discussion

This study shows that CBNAAT remains a valuable molecular tool for the rapid identification of TB in routine practice. The overall positivity (590/2640; 22.35%) among patients with presumptive TB is comparable with the 18%-30% range reported by Prakash et al. [6], Sidiq et al. [7], and Mustafa et al. [8]. The percentage of reports varies by region, most likely owing to variations in disease prevalence, patient characteristics, specimen mix, and referral practices. CBNAAT is also useful for early clinical decision-making because of its ability to provide microbiological confirmation rapidly and, at the same time, identify rifampicin resistance. The rate of positivity was much higher for pulmonary specimens than for extrapulmonary specimens. This is a natural occurrence; respiratory specimens tend to have more bacilli than specimens taken from other parts of the body, and extrapulmonary disease is typically paucibacillary and may involve anatomic locations that are inaccessible. This has been described in specimens of other types [9,10]. Although extrapulmonary disease may have a lower positivity rate, CBNAAT is still useful due to the significant reduction in diagnostic delay when compared to traditional methods [4,5].

Male sex was independently associated with CBNAAT positivity. Similar associations have been seen in high-burden settings such as India [6-8]. Occupation, smoking, alcohol consumption, late health-seeking, and behavioral factors may account for some of the increased burden among males. The higher positivity in males found in our study suggests the importance of active case-finding in men in the adult age group [11].

In addition, being rural was independently linked to CBNAAT positivity. Rural populations may lack access to healthcare, may receive delayed diagnosis, may be overcrowded, may be disproportionately undernourished, and may have lower socioeconomic status, all of which can sustain transmission [12]. The expansion of decentralized molecular diagnostic services and community-based screening could facilitate early diagnosis, and treatment delays could be avoided [12].

Diabetes mellitus was independently associated with higher chances of CBNAAT positivity in the present study. Diabetes has been shown to affect innate immunity and adaptive immunity by affecting the function of macrophages, changing the production of cytokines, and decreasing cell-mediated response [13]. Hyperglycemia may allow the survival of mycobacteria and help slow their clearance, making individuals more susceptible to active disease. This implies the need for routine, two-way screening for TB and diabetes that is currently advocated by national and international programs [11,13].

HIV infection was also independently associated with CBNAAT positivity. Progressive depletion of CD4-positive T lymphocytes compromises host defense and increases the risk of both primary disease and reactivation of latent infection; therefore, the integration of TB and HIV diagnostic services remains essential to enable early diagnosis, reduce morbidity, and improve outcomes [14].

Previous ATT was independently associated with CBNAAT positivity in the present study. Patients with a previous treatment history are more likely to have a relapse, treatment failure, or recurrent disease [14]; therefore, they may have a higher probability of microbiologically confirmed TB. Previous treatment is also a well-known risk factor for DR-TB due to previous exposure to ATT drugs. These findings support the present NTEP recommendations that emphasize the importance of routine molecular testing among previously treated patients to allow for early diagnosis and detection of rifampicin resistance [15].

Rifampicin resistance was identified in 5.76% (n = 34) of confirmed cases, comparable with reports from other Indian centers [6-8]. However, regional differences are expected owing to variation in prior treatment exposure, local MDR-TB burden, referral patterns, and adherence. Early molecular identification of rifampicin resistance may enable prompt referral for confirmatory drug-susceptibility testing and timely initiation of appropriate second-line therapy, thereby limiting transmission of resistant strains [13,16].

Our findings are consistent with current WHO and NTEP recommendations [1-3,15], which advocate for quick molecular diagnostic tests as the initial diagnostic test for patients suspected of having TB so as to enable early microbiological confirmation and simultaneous identification of rifampicin resistance. The fact that rifampicin-resistant TB was present in 5.76% (n = 34) of CBNAAT-positive cases in our research population highlights the ongoing need for routine molecular drug-resistance testing in high-burden settings. Even though direct comparison of CBNAAT-positive cases with national surveillance estimates is limited because of differences in research populations and reporting methods, our results support the continued use of CBNAAT in conventional diagnostic protocols for quick diagnosis and early identification of DR-TB.

Among extrapulmonary specimens, CBNAAT positivity rates varied considerably. Lymph node and pus/abscess aspirates showed the highest yields, while tissue biopsy and gastric aspirate showed the lowest. These patterns are largely consistent with previous studies that reported that tissue-based and aspirate specimens show a higher diagnostic yield than fluid specimens [6,7,12].

An important strength of this study is that a large number of specimens and confirmed TB cases were examined in a defined population in a single geographic region, which increases the internal consistency of the associations found and gives reliable region-specific estimates of CBNAAT performance for the Vindhya area. Also, the use of multivariable logistic regression to identify factors independently associated with CBNAAT positivity after adjustment for confounding; the associations observed were clinically coherent and consistent with current understanding of TB epidemiology [17] and may help clinicians prioritize high-risk groups for targeted screening.

Limitations

The retrospective, single-center design may limit generalizability. The observed positivity rate could not be taken as the prevalence of TB in the general population because the study only included clinically suspected TB patients who were referred for CBNAAT under routine programmatic settings. Culture and comprehensive phenotypic drug-susceptibility testing were not available for all patients, precluding formal assessment of diagnostic accuracy metrics against a microbiological reference standard, including sensitivity, specificity, positive predictive value, and negative predictive value. Therefore, rather than focusing on the diagnostic accuracy of CBNAAT, the results should be regarded as representing its clinical value in everyday practice. Treatment outcomes and long-term follow-up were beyond the study’s scope. The related adjusted odds ratios should be viewed cautiously because the variance inflation factors for age and history of TB contact were more than 5 (7.35 and 5.89, respectively), suggesting some shared variation among these variables. The true TB burden may be underestimated in the absence of culture, especially in paucibacillary extrapulmonary cases where CBNAAT sensitivity is known to be lower. Information regarding subsequent empirical treatment, alternative diagnostic pathways, or clinical outcomes for patients who failed to provide adequate specimens was not available from the retrospective records and therefore could not be evaluated. Among extrapulmonary specimens, sample sizes in many individual categories were small (e.g., ascitic fluid, bone/joint aspirate, gastric aspirate), and hence these findings should be interpreted in a descriptive manner rather than as definitive diagnostic performance data. Prospective multicenter studies incorporating culture, GeneXpert Ultra, and outcome data would allow a more comprehensive assessment of diagnostic performance.

## Conclusions

This study provides real-world evidence supporting the clinical utility of CBNAAT for the rapid microbiological confirmation of pulmonary and extrapulmonary TB in the high-burden Vindhya region of Central India. Male sex, rural residence, diabetes mellitus, HIV infection, previous TB treatment, and type of pulmonary specimen were all independently associated with CBNAAT positivity, which was higher in pulmonary specimens than extrapulmonary specimens. These findings are consistent with current NTEP recommendations advocating universal access to rapid molecular diagnostics for timely diagnosis and effective TB control. Prospective, multicentric studies using treatment outcome data are required to confirm the impact of these findings on broader TB control strategies and efforts.
